# Positive Outcomes of Physiotherapy in a Post-operative Case of Squamous Cell Carcinoma of Tongue and Buccal Mucosa Along With Transfemoral Amputation

**DOI:** 10.7759/cureus.50435

**Published:** 2023-12-13

**Authors:** Komal S Mandhane, Priyanka A Telang, Jaee P Kapre

**Affiliations:** 1 Community Health Physiotherapy, Ravi Nair Physiotherapy College, Datta Meghe Institute of Higher Education and Research, Wardha, IND

**Keywords:** rehabilitation, physical therapy, transfemoral amputation, buccal mucosa, tongue cancer, oral cancer

## Abstract

Oral cancer is a type of malignant neoplasia that develops in the oral cavity or lips and is commonly referred to as squamous cell carcinoma (SCC) because of its histopathology. In this report, we present to you a case of a 35-year-old male patient operated on for moderately differentiated SCC of the lateral border of the tongue and right buccal mucosa with a two-year-old case of trans-femoral amputation. Postoperatively, the patient faced issues with breathing, mouth opening and closing limitations, and painful movements of the neck and right shoulder. An intensive physiotherapy care regimen was designed and consistently implemented for two weeks to tackle the surgical results that were compromising the patient's quality of life. At the two-week evaluation, enhancements in tongue movement, mouth opening, shoulder and cervical joint movement, thoracic mobility, lower limb strength, and gait were seen, confirming the efficacy of the intended therapy. The patient was assessed before and after the rehabilitation using range of motion, manual muscle testing, the Mallampati scale, the numerical pain rating scale, the amputee mobility predictor assessment tool, and the Royapettah scoring system.

## Introduction

Oral cancer is a type of malignant neoplasia that develops in the oral cavity or lips and is commonly referred to as squamous cell carcinoma because of its histopathology. Oral cavity cancers are one of the most common cancers in India, and The oral cavity is recognized as the sixth to ninth most prevalent anatomical site for carcinoma, depending mostly on the nation (and occasionally even geographic region within specific countries) and gender of the patient populations [1]. Drinking alcohol, chewing kharra, and tobacco are all known to increase the chance of developing oral squamous cell cancer [2]. The most prevalent form of oral cancer is squamous cell carcinoma (SCC) of the tongue. Most occurrences affect the tongue's lateral edge and, very rarely the dorsum [3]. Clinically, the cases of tongue SCCs in individuals under 45 often originate from the tongue's periphery, as well as in people who might not be smokers or drinkers [4]. Many researchers have historically viewed young adult tongue SCCs as fairly aggressive clinical manifestations, with a higher probability of loco-regional relapse, lower survival rates than the regular populace, and a consequent requirement for an even more vigorous treatment strategy [5]. The excision of the primary tumor while preserving a substantial margin of healthy tissue in its wake is the cornerstone of surgical oncology for tongue tumors [6,7].

Lower-limb amputation makes it difficult for amputees to go about their daily lives. This causes issues like ambulation limitations, which lead to impaired mobility and a low quality of life (QoL) [8]. Amputees who lost a lower limb find it challenging to carry out their regular activities. Gait problems result from this, which reduces mobility and lowers QoL. Since the knee joint is lost in patients with transfemoral (above knee) amputations, it has been well-known that such people require more energy to walk normally [9]. The mechanical disadvantage of the remnant femur being in an abducted posture requires the patient to walk more energetically even with a prosthesis that fits them well, which contributes to the gait disruption in patients with transfemoral amputations.

Early-stage buccal SCC is presently treated with either surgery or irradiation as a single modality, but advanced cancers are advised to get postoperative radiation in addition to surgical removal [10]. The ill effects of surgery are well known. Infection, pain, swallowing difficulty, speech impairments, and other oral complications are common after any surgery. To restore normal lung volumes and capacities, improve mobility, improve activities of daily living, ensure early discharge, and prevent the formation of restrictive scar tissue, it is particularly critical to begin physiotherapeutic exercises in the early postoperative period. Restricted mouth opening, known as trismus, poses a significant challenge for individuals dealing with neck and head cancer. Approximately 2% of patients experience this due to tumor growth, while about 8% encounter it as a result of surgical or radiotherapy interventions. Restricting mouth openings can make it difficult to speak, laugh, yawn, or maintain good dental hygiene, which can impact one's QoL [11].

This study discusses the case of a male patient who underwent surgery for cancer of the right side buccal mucosa and lateral border of the tongue and is also a known case of the two-year-old above-knee amputation of the right lower limb. For a period of two weeks, the patient underwent a series of physical therapy exercises intended to enhance range of motion (ROM), strengthening, and effective breathing techniques.

## Case presentation

Patient information

This document outlines the case of a 35-year-old male who presented with a painful, non-healing ulcer on the lower right side of the jaw that persisted for 1.5 months. The ulcer initially measured small but expanded to approximately 4 x 3 cm. The patient experienced gradual onset, continuous, dull aching pain localized to the area, along with a burning sensation and difficulty. The patient gave a history of a road traffic accident (RTA) that caused a fracture of his left clavicle, which was surgically managed. In 2021, the patient again met with an RTA that caused a fracture of the right forearm, which was managed by open reduction and internal fixation. The patient also had to undergo transfemoral amputation of the right lower limb as he suffered from a severe crush injury chewing. Upon assessment in the oral surgery department, diagnostic procedures including contrast-enhanced computed tomography, blood count, and liver function tests were conducted, leading to a diagnosis of moderately differentiated squamous cell carcinoma affecting the lateral border of the tongue and right buccal mucosa. Surgical intervention was recommended, and the patient underwent composite resection of the lesion, modified radial neck dissection on the right side, segmental mandibulectomy, reconstruction using a pectoralis major myocutaneous flap, and a tracheostomy. Postoperatively, he complained of pain at the sulture site and difficulty performing neck movements. For the above complaints, the patient was referred to physiotherapy management.

Clinical findings

The patient's written and verbal consent was acquired. He was mesomorphic in build and was hemodynamically stable. On observation, the patient was seen in a sitting position with both elbows and shoulders in a neutral position with the hip flexed to 90° and the knee extended. On palpation, tenderness was found to be grade 2, that is, the patient complained of pain and winces. The right submandibular lymph node is firm and non-tender to the touch. The patient rated pain as 8/10 according to the numerical pain rating scale (NPRS).

Physiotherapeutic intervention

A well-planned physiotherapy (PT) regimen was comprehended for the betterment of the patient on postoperative day one, as given in Table 1.

**Table 1:** Physiotherapy rehabilitation reps: Repetitions; ROM: Range of motion; TMJ: Temporomandibular joint

Goals of physical therapy	Intervention	Duration and frequency
To avoid post-operative respiratory complications	Thoracic expansion exercises and diaphragmatic breathing exercises	10 reps three times a day
To relieve trismus	Mouth opening and closing exercises and active ROM exercises of the TMJ in all planes. Chin tucks. Mirror therapy.	10 reps twice a day
To improve speech	ROM of the lips, which included spreading them as far as possible. Lateral deviation of the mouth. Extending the tongue outward and retracting it backward. Vowel pronunciation.	10 reps twice a day after one week of operation
To improve and maintain cervical ROM	Active cervical flexion, extension, lateral flexion, and side flexion exercises	10 reps twice a day
To increase and maintain the strength of cervical musculature	Isometrics of the cervical flexors, extensors, lateral flexors, and rotators	10 reps twice a day with a 10-sec hold initially, later progressed to 30 secs
To improve and maintain shoulder ROM	Active shoulder flexion, extension, abduction, and adduction exercises. Codman’s pendular exercises. Finger ladder. Wand exercises	10 reps twice a day
To improve the strength of the affected shoulder	Strengthening with manual resistance initially, progressing to ½ liter water bottle and later on with 1-liter water bottle	10 reps twice a day
To relieve edema and avoid complications of immobilization	Ankle pumps of the unaffected lower limb. Heel slides of left lower limb. Limb elevation.	20 reps thrice a day
To increase the strength of the amputated lower limb	Strengthening of hip flexors and abductors with manual resistance	10 reps twice a day
To increase the strength of unaffected lower limb	Strengthening of hip, knee, and ankle musculature using weights	10 reps twice a day
To improve gait	Gait training with the help of a walker initially. Progressed to crutches. Lastly, using a cane	Two rounds around a 100-meter hallway

**Figure 1:**
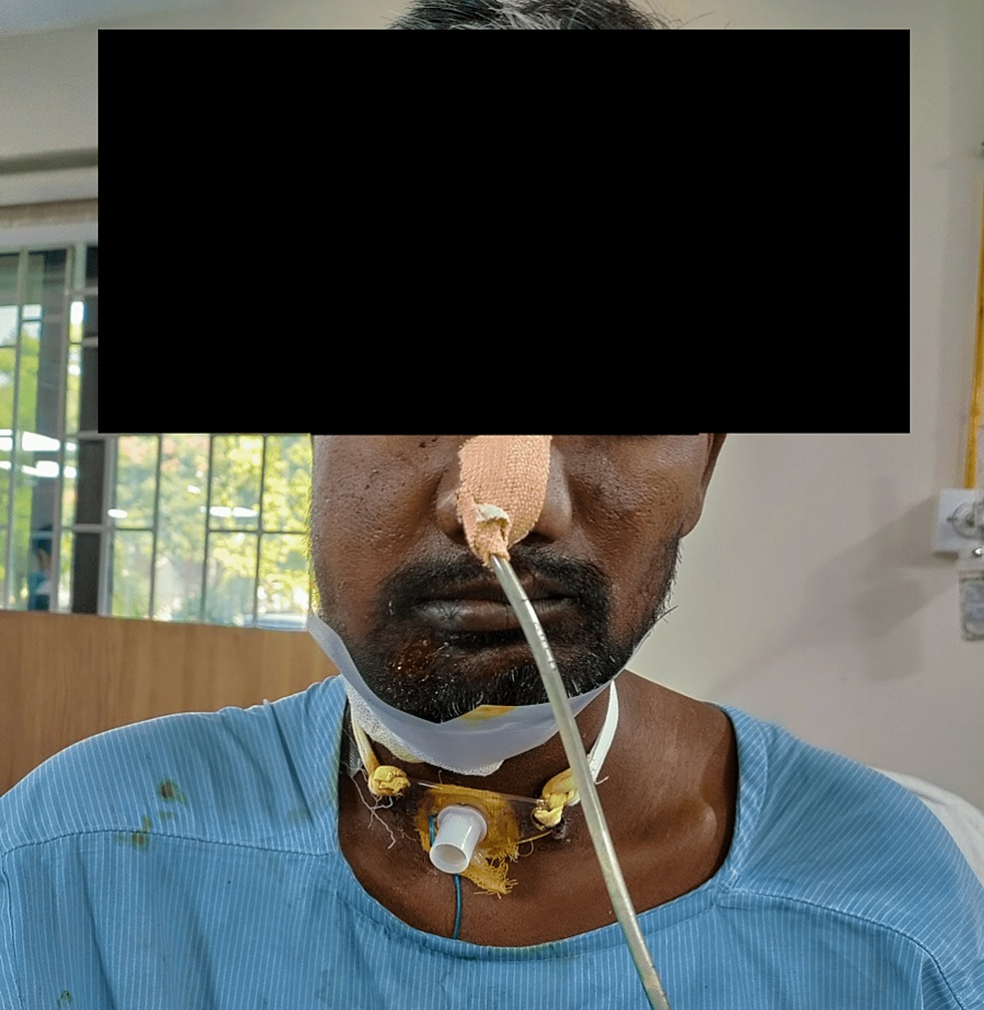
Patient performing mouth opening and closing exercise

The exercises were conducted under supervision in our super specialty in-patient department for two weeks. Figure 1 shows showing patient performing mouth closing and opening exercises. The patient was taught the home exercise program (HEP) on the day of discharge. Before initiating the treatment, the patient, his family, and caregivers were educated about the condition, surgery and its complications, benefits of exercise. In this circumstance, it was important to keep boosting the patient's self-confidence and encouraging him throughout the therapy.

**Figure 2:**
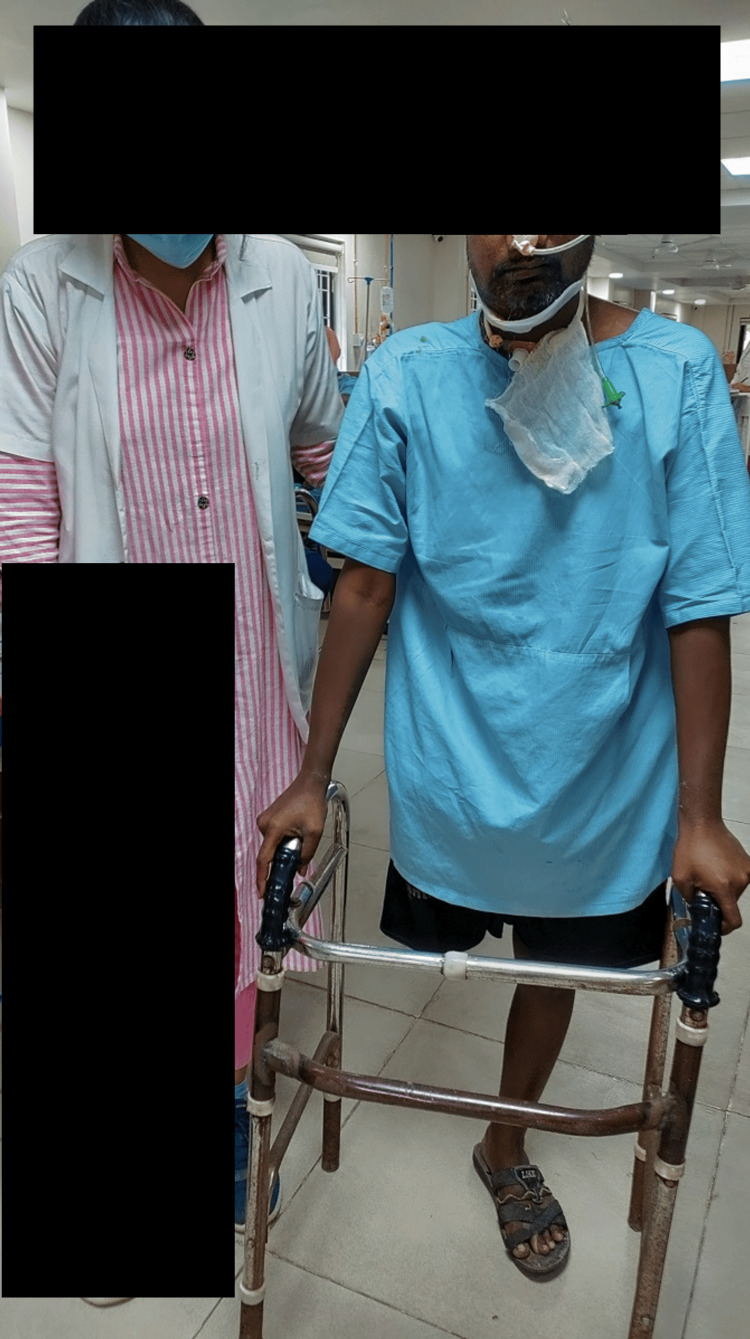
Gait training with walker

Figure 2 shows ambulation training given to the patient with the support of a walker under the observation of the therapist.

Outcome measures

The patient was assessed thoroughly using the NPRS, amputee mobility predictor assessment tool, ROM, manual muscle testing, and the Mallampati scale for mouth opening before and after the rehabilitation program (Table 2).

**Table 2:** Outcome measures assessed pre and post-rehab NPRS: Numerical pain rating scale; ROM: Range of motion; MMT: Manual muscle testing

Outcome measures	Components	Pre-rehab (postoperative day 1)		Post-rehab (postoperative day 14)	
NPRS		7/10		2/10	
Mallampati score		Grade IV (only the hard palate is visible)		Grade I (soft palate, uvula and pillars are visible)	
Amputee mobility predictor assessment tool		9/39		28/39	
-ROM (in degrees)					
Cervical joint	Flexion	0-30		0-40	
	Extension	0-35		0-45	
		Right side	Left side	Right side	Left side
	Side flexion	0-15	0-19	0-20	0-21
	Lateral rotation	0-35	0-40	0-40	0-48
Shoulder joint	Flexion	0-80	0-90	0-160	0-175
	Extension	0-30	0-40	0-45	0-50
	Abduction	0-70	0-100	0-165	0-175
	Adduction	70-0	100-0	165-0	175-0
-MMT	Flexors	3/5		4/5	
Cervical joint	Extensors	3/5		4/5	
	Side flexors	3/5	3+/5	3+/5	4/5
Shoulder joint	Flexors	3/5	3+/5	4/5	5/5
	Extensors	3+/5	3+/5	5/5	5/5
	Abductors	3/5	3+/5	4+/5	5/5
	Adductors	3/5	3+/5	5/5	5/5
	Medial rotators	3+/5	3+/5	5/5	5/5
	Lateral rotators	3/5	3+/5	5/5	5/5
Hip joint	Side flexors	3+/5	3+/5	4/5	4/5
	Flexors	3/5	3+/5	4/5	4/5
	Extensors	3+/5	3+/5	4/5	4/5
	Abductors	3/5	3+/5	4/5	4/5
	Adductors	3+/5	3+/5	4/5	4/5

## Discussion

One of the most prevalent cancers in our nation is head and neck cancer, and 30% of all cases of this disease occur in the oral cavity. The head and neck portion, which is the most significant body component in terms of appearance, also serves as the foundation for vital basic bodily activities, including chewing, swallowing, and communication. The management of oral cancers is primarily through radiotherapy, chemotherapy, and surgery that includes resection of the lesion, mandibulectomy, and skin grafting. Surgery likely contributes to functional and cosmesis-related morbidities that range from tolerable to disastrous. Mouth opening, oral competence, occlusion, speech, and swallowing are impacted functions. Physiotherapy is important in the rehabilitation of patients with oral cancer who are undergoing different therapies such as mouth-opening exercises with Therabite devices, neck and head exercises, and mobility of the shoulder [12].

In this instance, a tailored PT protocol was incorporated for the patient. These included exercises that helped prevent postoperative respiratory complications, improve temporomandibular joint, cervical, and shoulder mobility, strengthen affected musculature, provide gait training, and improve the QoL of the patient. The patient's adaptation to the rehabilitation training greatly improved chewing and swallowing. To make the patient independent by enabling him to do his basic daily activities, the patient was encouraged and motivated throughout the sessions. Appropriate gait training using a walker was initiated from day one of admission, as the patient couldn't afford a prosthesis. The patient's family was asked to construct railings or bamboo supports in the house to aid during mobilization so that the patient could carry out his daily activities without any help.

Tacani et al. did a study on the effect of physical therapy on 32 patients with head and neck cancer. Physical therapy was successful in lowering discomfort and lymphedema [13]. Zuydam et al. conducted a study on patients undergoing surgical resection of the oropharynx, including the base of the tongue, to identify the effectiveness of compensatory procedures and therapy techniques in eliminating aspiration. The techniques included chin tuck and supraglottic swallow and were found to be helpful in 50% of patients [14]. Another study by Logemann and others demonstrated the effect of ROM exercises for jaw, tongue, swallowing, and speech rehabilitation on postoperative patients [15].

While the surgeons were planning the discharge of the patient, the HEP was taught to him. It consisted of the same in-patient program along with other exercises like moving the jaw in a circular motion as far as possible, bubble-blowing exercises, smiling as wide as possible, and water-holding exercises. The patient was asked to perform these exercises for 10-15 minutes three times a day. The patient was called for a follow-up after two months of discharge. Then he was assessed using the Royapettah scoring system, where the score was 7/35 (poor) on postoperative day one, which progressed to 31/35 (excellent). The limitations of our study include the fact that long-term follow-up was not able to be done.

## Conclusions

The outcomes of oral cancer treatment in terms of appearance, functionality, and mental impact could be devastating for patients. A non-functional limb has a huge impact on a patient's QoL and independence. The main goal of treatment in a patient with carcinoma of the tongue and buccal mucosa with transfemoral amputation is to improve the patient's mobility and strength, functional independence, and incorporate a sense of self-esteem to go back to his normal life. There was a notable impact and improvement in the patient in the span of two months which included two weeks of in-patient therapy and two months of HEP. The outcome measures and our patient's personal experience regarding the benefits of exercise proved that the therapy was helpful overall.

## References

[1] Pires FR, Ramos AB, Oliveira JB, Tavares AS, Luz PS, Santos TC (2013). Oral squamous cell carcinoma: clinicopathological features from 346 cases from a single oral pathology service during an 8-year period. J Appl Oral Sci.

[2] Fang QG, Shi S, Li ZN, Zhang X, Liua FY, Xu ZF, Sun CF (2013). Squamous cell carcinoma of the buccal mucosa: analysis of clinical presentation, outcome and prognostic factors. Mol Clin Oncol.

[3] Okubo M, Iwai T, Nakashima H (2017). Squamous cell carcinoma of the tongue dorsum: incidence and treatment considerations. Indian J Otolaryngol Head Neck Surg.

[4] Ren ZH, Gong ZJ, Wu HJ (2017). Unit resection of buccal squamous cell carcinoma: description of a new surgical technique. Oncotarget.

[5] Paderno A, Morello R, Piazza C (2018). Tongue carcinoma in young adults: a review of the literature. Acta Otorhinolaryngol Ital.

[6] Randall CJ, Shaw HJ (1986). Malignant tumours of the tongue in young adults. Experience of a secondary referral centre. J Laryngol Otol.

[7] Hui H C, Tung S C, Ping G L (2007). Clinicopathologic evaluation of prognostic factors for squamous cell carcinoma of the buccal mucosa. Jour of the Chinese Med Asso.

[8] Ichimura D, Hisano G, Murata H, Kobayashi T, Hobara H (2022). Centre of pressure during walking after unilateral transfemoral amputation. Sci Rep.

[9] Dite W, Connor HJ, Curtis HC (2007). Clinical identification of multiple fall risk early after unilateral transtibial amputation. Arch Phys Med Rehabil.

[10] Casper C, Peter C.M. de W, Lucas A. P (2022). Treatment results of patients with a squamous cell carcinoma of the buccal mucosa. Oral Oncol.

[11] DallAnese AP, Schultz K, Ribeiro KB (2010). Early and long-term effects of physiotherapy for trismus in patients treated for oral and oropharyngeal cancer. Appl Cancer Res.

[12] Renu P, Diana MO (2019). Postoperative physiotherapy management for complications related to cancer of buccal mucosa (head and neck cancer). Indian J Physiother Occup Ther.

[13] Tacani RE, Machado AFP, Goes JCGS (2014). Physiotherapy on the complications of head and neck cancer: retrospective study. Int J Head Neck Surg.

[14] Zuydam AC, Rogers SN, Brown JS, Vaughan ED, Magennis P (2000). Swallowing rehabilitation after oro-pharyngeal resection for squamous cell carcinoma. Br J Oral Maxillofac Surg.

[15] Logemann JA, Pauloski BR, Rademaker AW, Colangelo LA (1997). Speech and swallowing rehabilitation for head and neck cancer patients. Oncol.

